# Tcf7l2 plays pleiotropic roles in the control of glucose homeostasis, pancreas morphology, vascularization and regeneration

**DOI:** 10.1038/s41598-017-09867-x

**Published:** 2017-08-29

**Authors:** Nicola Facchinello, Estefania Tarifeño-Saldivia, Enrico Grisan, Marco Schiavone, Margherita Peron, Alessandro Mongera, Olivier Ek, Nicole Schmitner, Dirk Meyer, Bernard Peers, Natascia Tiso, Francesco Argenton

**Affiliations:** 1 Department of Biology, University of Padova, I-35131 Padova, Italy; 2 Laboratory of Zebrafish Development and Disease Models, GIGA-R, University of Liege, B-4000 Sart Tilman, Belgium; 3 Department of Information Engineering, University of Padova, I-35131 Padova, Italy; 4 Institute of Molecular Biology, CMBI, Leopold-Franzens-University Innsbruck, A-6020 Innsbruck, Austria

## Abstract

Type 2 diabetes (T2D) is a disease characterized by impaired insulin secretion. The Wnt signaling transcription factor *Tcf7l2* is to date the T2D-associated gene with the largest effect on disease susceptibility. However, the mechanisms by which TCF7L2 variants affect insulin release from β-cells are not yet fully understood. By taking advantage of a *tcf7l2* zebrafish mutant line, we first show that these animals are characterized by hyperglycemia and impaired islet development. Moreover, we demonstrate that the zebrafish *tcf7l2* gene is highly expressed in the exocrine pancreas, suggesting potential bystander effects on β-cell growth, differentiation and regeneration. Finally, we describe a peculiar vascular phenotype in *tcf7l2* mutant larvae, characterized by significant reduction in the average number and diameter of pancreatic islet capillaries. Overall, the zebrafish Tcf7l2 mutant, characterized by hyperglycemia, pancreatic and vascular defects, and reduced regeneration proves to be a suitable model to study the mechanism of action and the pleiotropic effects of Tcf7l2, the most relevant T2D GWAS hit in human populations.

## Introduction

The world prevalence of diabetes is estimated to be 9% among the adult population (aged 20–79 years). Diabetes mellitus is a disease of metabolic dysregulation; initially diagnosed as hyperglycaemia, ultimately results in blood vessel defects that lead to various complications such as cardiovascular disease and stroke, retinopathy, nephropathy, neuropathy, and impaired wound healing. Relative or absolute deficiency of insulin-producing β cells in pancreatic endocrine islets underlies the pathogenesis of both type 1 and type 2 diabetes mellitus (T1D and T2D, respectively). Thus, there is considerable interest in understanding the signalling mechanisms that stimulate pancreatic islet cell growth and differentiation.

A genome-wide association study (GWAS) performed in 2006 first identified a link between TCF7L2 polymorphisms and the risk of T2D among European and American populations^1^. Among common genetic lesions linked to T2D, polymorphisms in the *TCF7L2* gene provide the strongest association with the disease manifestation. This gene encodes for a transcription factor, which, like other members of the TCF/LEF family, interacts with β-catenin as a downstream effector of the Wnt signalling pathway. Wnt proteins are a family of highly conserved secreted proteins that regulate multiple developmental processes, including proliferation of organ-specific stem/progenitor cell populations, tissue growth and patterning, and cell fate determination in diverse ontogenetic processes. Interestingly, many studies point to a fundamental role for the Wnt pathway in β-cell biology, but more research is needed to determine whether this molecular signalling is active in β cells *in vivo* ^2^.

As far as concerns the specific expression and functional role of the Wnt signalling effector Tcf7l2 in β cells, current studies are not yet fully conclusive. In the work of Columbus *et al*.^3^ qRT-PCR analysis has been carried out to analyse *Tcf7l2* expression in rodent gut, pancreas, isolated pancreatic islets, and cultured cell lines. The expression of *Tcf7l2* was relatively lower in the pancreas compared to the gut, and the immuno-staining failed to detect *Tcf7l2* signals in mouse islets^3^. Functional studies on *Tcf7l2* in murine models and humans have indicated that individuals with TCF7L2 polymorphisms exhibit impaired insulin secretion and enhanced rate of hepatic glucose production^4–7^. However, whether *Tcf7l2* directly regulates the function of β-cells remains controversial^8–10^. For instance, Boj *et al*. conclude that *Tcf7l2* is not important for β-cell function in mice but it rather controls the hepatic response to perinatal and adult metabolic demand^11^. On the other hand, other studies report altered β-cell formation and function upon genetic depletion of *Tcf7l2* during pancreas development^12–16^.

Given the existing controversy in the literature over the relative importance of *Tcf7l2* for proper development of β cells, liver and/or other tissues, as well as the contributions of extra-pancreatic tissues to risk variants activity on diabetes susceptibility^17^, the present study has been designed to investigate the general effects of *Tcf7l2* in a simple model: the zebrafish (*Danio rerio*). This vertebrate organism offers numerous advantages that have been exploited in this study. In particular, the accessibility and optical clarity of the zebrafish embryos allow cell manipulation and sophisticated *in vivo* imaging approaches that would be far more difficult in a mammalian model. Importantly, the zebrafish pancreas shares important similarities with its human counterpart. In both species the pancreas consists of an exocrine compartment (formed by acinar cells), producing digestive enzymes secreted into a ductal system and transported to the digestive tract, and an endocrine compartment, represented by islets embedded in a dense capillary network, playing a critical role in blood sugar homeostasis^18^.

By taking advantage of an available zebrafish *tcf7l2* mutant (previously named *tcf4*), bearing a functionally null allele^19^, we present here a detailed analysis on the role of *Tcf7l2* in glucose homeostasis and in the development, regeneration and vascularization of the pancreatic islet.

## Results

### Postprandial increase of blood glucose in heterozygous *tcf7l2*^*exI*/+^ adults

In order to prove the suitability of zebrafish as a model for *Tcf7l2*-dependant Type 2 Diabetes (T2D), we analysed the blood glucose levels in a mutant background. Specifically, we focussed on the zebrafish *tcf7l2* ^*exI*^ mutation, which was isolated from a zebrafish library mutagenized with N-ethyl-N-nitrosourea (ENU)^19^. The *tcf7l2* ^*exI*^ allele carries a G-to-A substitution within the splice donor site of intron 1; this mutation leads to intron retention and formation of a short-truncated protein. Notably, *tcf7l2* ^*exI*/*exI*^ homozygous mutants are known to have an intestinal phenotype due to proliferation defects^19^. Only less than 1% of homozygous *tcf7l2* ^*exI*/*exI*^ fish can reach the adult stage, as most of them die by 6 weeks post fertilization. Therefore, in this study, all experiments performed to evaluate the role of *tcf7l2* during adulthood have been carried out on *tcf7l2* ^*exI*/+^ heterozygous mutants.

In order to characterize the metabolic phenotype of *tcf7l2* heterozygotes, we performed three sets of experiments in which we measured changes of blood glucose concentration during different dietary conditions by using the protocol of Eames and colleagues^20^.

**Figure 1:**
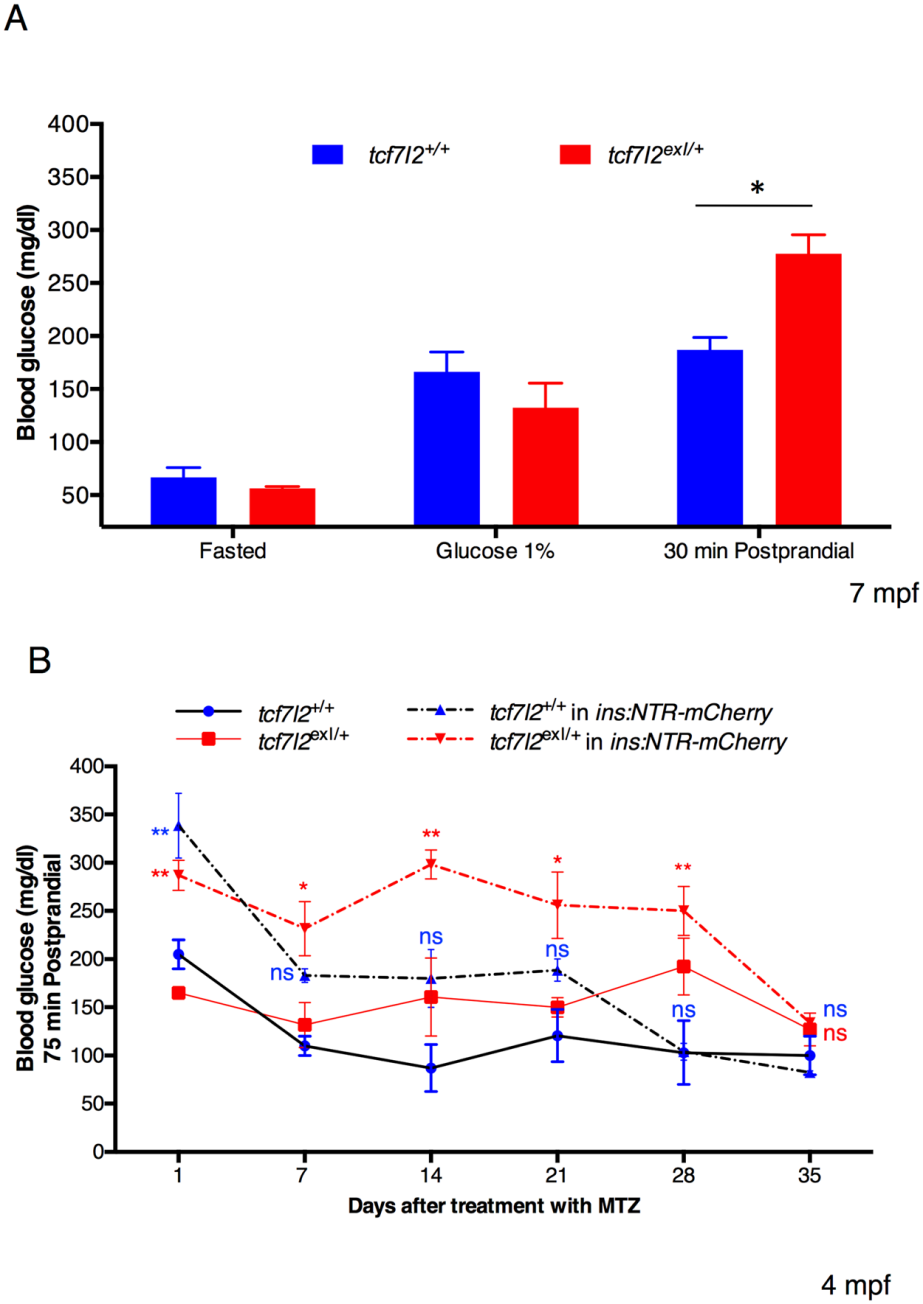
Postprandial increase of blood glucose in heterozygous *tcf7l2* ^*exI*/+^ adults. Blood glucose levels in wild type and *tcf7l2* ^*exI*/+^ adult fish (7 mpf) under different dietary conditions (Fasted, 1% Glucose and 1 h Postprandial). Data were obtained from five fishes per genotype, repeated in 2 different experiments. Average values are given for each sample and standard errors of the mean are indicated; *p < 0.05. (**B**) Blood glucose levels after 75 minute postprandial in wild type and *tcf7l2* ^*exI*/+^ adult fish (4 mpf), monitored during recovery after MTZ treatment. Values represent the mean ± SEM. Red asterisks indicate that blood glucose levels of *tcf7l2* ^*exI*/+^ mutants in *ins:NTR-mCherry* transgenic background are significantly different from *tcf7l2* ^+/+^ controls; **p < 0.01 and *p < 0.05; n = 5 individuals per genotype. Blue “ns” (not significant) indicates no statistical difference between *tcf7l2* ^+/+^ in *ins:NTR-mCherry* transgenic background and *tcf7l2* ^+/+^ controls at 7, 14, 21, 28 and 35 days after treatment with MTZ.

In the first dietary condition, we measured changes in glucose level in the absence of food by fasting *tcf7l2* ^*exI*/+^ heterozygous fish for 4 days. After 4-day fasting we could not detect differences in the glycaemia between wild type and *tcf7l2* ^*exI*/+^ individuals. In the second dietary condition we induced hyperglycaemia by placing zebrafish in a 1% glucose solution; as already reported by Gleeson and colleagues^21^, the immersion in a glucose solution resulted in increased blood glucose without any statistically significant difference between wild type and *tcf7l2* ^*exI*/+^ fish (Fig. 1A). However, despite constant immersion in 1% glucose, the glycaemic peak is transient and blood glucose levels stabilize to values higher than normal^21^. For this reason, we chose to analyse the blood glucose level after 4 days of continued immersion in 1% glucose. Nonetheless, also in this condition, blood glucose level was similar between *tcf7l2* ^+/+^ controls and *tcf7l2* ^*exI*/+^ heterozygotes. Finally, in the third dietary condition we determined changes in glucose levels 30 minutes postprandial. In this latter test, we could reveal a significant increase of blood glucose level in *tcf7l2* ^*exI*/+^ heterozygous animals compared with *tcf7l2* ^+/+^ controls (Fig. 1A) indicating that in zebrafish, under specific nutritional contexts, partial loss of *tcf7l2* activity can lead to an increase of blood glucose, a condition that somehow mimics T2D.

Next, we applied a novel, non-lethal and reliable method for repeated blood collection from adult zebrafish^22^, and combined it with a β-cell ablation strategy in order to evaluate, from glucose metabolism data, the regenerative capacity of the β-cell mass in heterozygous *tcf7l2* ^*exI*/+^ mutants and wt controls.

Wt and *tcf7l2* ^*exI*/+^ adults in *Tg*(*ins:NTR-mCherry*) background were treated with 10 mM Metronidazole (Mtz) for 24 hours^23^ leading to a severe hyperglycaemia. To investigate the regenerative capacity of the pancreatic islet after NTR/Mtz-mediated ablation, we measured the blood glucose in wt and *tcf7l2* ^*exI*/+^ fish at different time points (Fig. 1B). In control adults, we observed a significant decrease in blood glucose only 7 days after treatment, indicating a recovery of β cells function, and normalization of glucose levels at 28 days. In contrast, Mtz-treated *tcf7l2* ^*exI*/+^ animals showed high glucose levels three weeks after Mtz treatment and displayed full recovery at 35 days (Fig. 1B).

### Canonical Wnt signalling and *tcf7l2* expression in the pancreas

**Figure 2:**
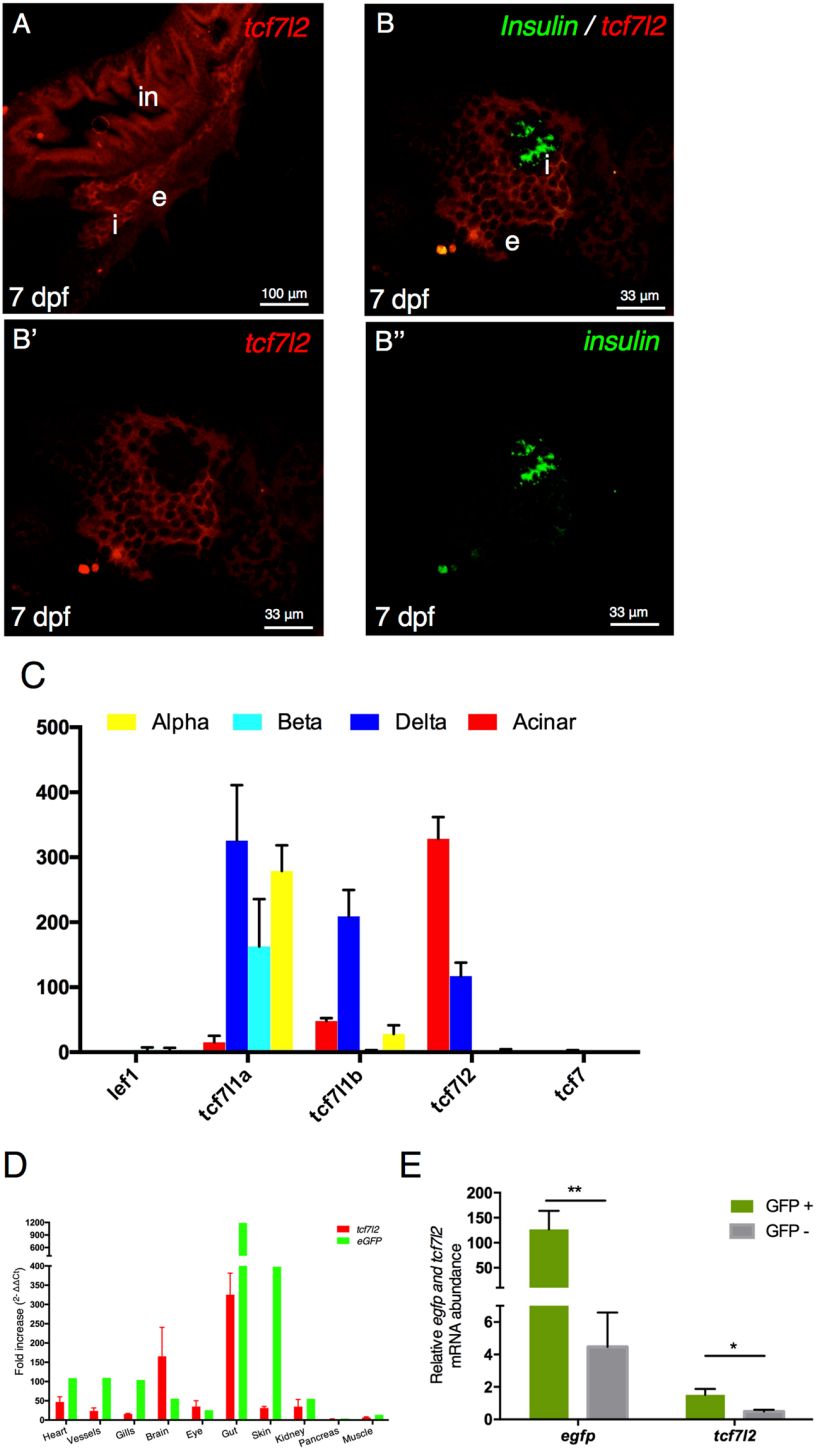
Canonical Wnt signalling and *tcf7l2* expression in the pancreas. (**A**,**B**) Single and double fluorescent WISH comparing the expression of *tcf7l2* and *insulin* at 7 dpf. Images show confocal single planes at 7 dpf at different magnification of (**A**) (20x) and (**B,B’,B”**) (60x). Merging the green and red channels identifies regions with distinct gene expression of the two genes (**B’,B”**). in: intestine, e: exocrine tissues, i: principal islet. Scale bar = 100 μm. (**C**) Expression level (count normalized by library size) obtained by RNAseq of different Tcf/Lef genes in α, β, δ and acinar cells from adult zebrafish. (**D**) *tcf7l2* and *egfp* expression values from 10 different tissues derived from transgenic *Tg*(*fli1a:EGFP*)^*y1*^ adult fish. Brain (Br), Eye (Ey), Gills (Gi), Gut (Gu), Heart (He), Kidney (Ki), Muscle (Mu), Pancreas (Pa), Vessels (Ve). (**E**) Enrichment of GFP^+^ cells from *Tg*(*fli1a:EGFP*)^*y1*^ zebrafish embryos. Relative expression of indicated genes, in GFP^+^ (green bars) and GFP^−^ (grey bars) cells, determined by quantitative RT-PCR. Relative expression levels were determined by normalization to *arp*. Values represent the mean ± SEM. Asterisk above column indicate statistical differences among groups.

Given this identified association between Tcf7l2 haplo-insufficiency and hyperglycaemia, we set out to investigate the potential role of canonical Wnt signalling, of which Tcf7l2 is a downstream effector, in endocrine pancreas development and/or function. The main endocrine islet of the zebrafish pancreas is embedded in the head of exocrine tissue^24, 25^. To detect canonical Wnt signalling activation in the endocrine pancreas, we crossed the *Tg*(*7xTCFXla*.*Siam:nlsmCherry*)^*ia5*^ strain, a reporter line for *in vivo* TCF/β-catenin activity^26^, with *Tg*(*ptf1a:EGFP*), a transgenic line that specifically labels the exocrine tissues of the developing pancreas. In this way, we could detect *in vivo* a weak expression of canonical Wnt signalling in the endocrine islet from 9 dpf (Supplementary Figure 1). Then, we analysed *tcf7l2* and *insulin* expressions by double fluorescent whole mount *in situ* hybridization. In our conditions, we could not detect the expression of *tcf7l2* in β cells at any of the considered developmental stages (72 hpf, 4 dpf and 7 dpf), while its expression was significant in the exocrine pancreas and intestine (Fig. 2A,B). This is in agreement with published data on the function of *tcf7l2* in zebrafish^19^. However, to rule out the possibility of low *tcf7l2* expression levels, below the whole-mount *in situ* hybridization detection threshold, we analysed its expression in different pancreatic cell types by RNAseq. The expression level of *tcf7l2* and other members of the TCF/LEF gene family (*tcf7l1a*, *tcf7l1b*, *tcf7* and *lef1*) was obtained from RNAseq datasets prepared from purified pancreatic endocrine cells (β-, α- and δ-cells) as well as from the exocrine acinar cells isolated from adult zebrafish^27^.

As shown in Fig. 2C, *tcf7l2* is expressed in acinar and at lower levels in δ-cells while no expression was detected in β and α cells. Additionally, *tcf7l1a* is mainly expressed in the three endocrine cell types while *tcf7l1b* is mainly detected in δ cells. Finally, *lef1* and *tcf7* were not detected in any of the considered pancreatic cell types. These results confirmed that *tcf7l2* is not expressed in β cells but only in δ and acinar cells.

As far as concerns the possible expression of *tcf7l2* in vascular cells, we could not detect significant signals in these cell types by whole-mount *in situ* hybridization, although RT-PCR could detect a good correlation (R^2^ = 79%) between the amplification of *tcf7l2* transcripts and the amount of endothelial-specific GFP in a set of considered samples (transgene: *Tg*(*fli1a:EGFP*)^*y1*^) (Fig. 2D). This observation has been further validated by quantitative PCR performed on GFP^+^ and GFP^−^ cells sorted from dissociated 6-dpf embryos of the *Tg*(*fli1a:EGFP*)^*y1*^ strain, a transgenic line fluorescently labelling endothelial cells (and, to a lesser extent, hematopoietic and pharyngeal arc cells)^28^. This analysis demonstrated that GFP^+^ cells are also positive to *tcf7l2* (Fig. 2E). All together, these results support the hypothesis that *tcf7l2* might be expressed in endothelial cells, thus exerting a potential role also in blood vessels.

### *tcf7l2* is required to regulate insulin expression and exocrine pancreas formation in *tcf7l2*^*exI*/*exI*^ homozygous mutants

**Figure 3:**
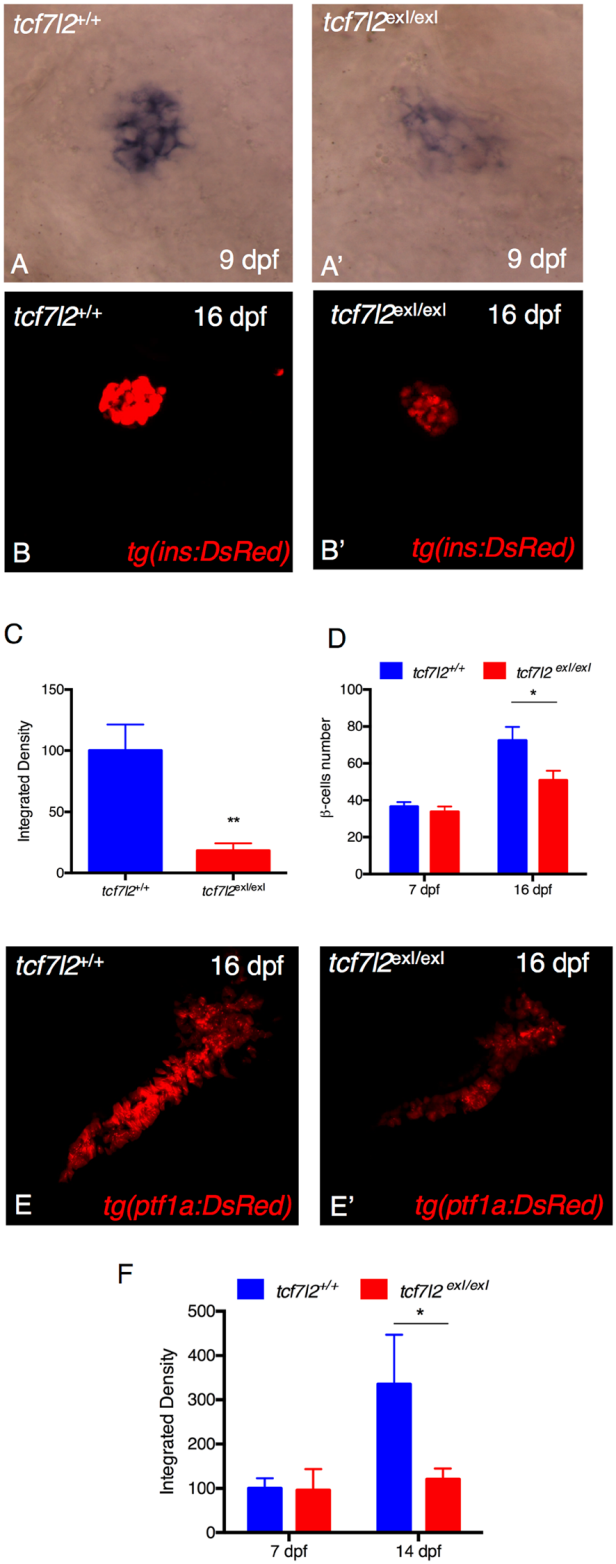
*tcf7l2* is required for regulation of insulin expression and exocrine pancreas development. Analysis of *insulin* in control embryos (**A**) and in *tcf7l2* ^*exI*/*exI*^ mutants (**A’**) by *in situ* hybridization. Lateral views of the pancreatic area are shown with the anterior side to the right. The expression of insulin is significantly reduced in the mutants. (**B–B”**) Analysis of pancreatic islet in *tcf7l2* ^*exI*/*exI*^ mutant in *Tg*(*ins:dsRed*) background. 2D projections of confocal Z-series images of DsRed expression in living *Tg*(*ins:DsRed*) embryos at 16 dpf. (**B**) wt; (**B’**) homozygous mutant. (**C**) Graphic presentation of the integrated density of fluorescence in the red channel in *tcf7l2* ^*exI*/*exI*^ mutant and wild-type sib controls in *Tg*(*ins:dsRed*) at 16 dpf. (**D**) Quantification of the number of β cells during juvenile growth of *tcf7l2* ^*exI*/*exI*^ and control siblings. (**E–E’**) Analysis of exocrine pancreas in *tcf7l2* ^*exI*/*exI*^ mutant in *Tg*(*ptf1a:dsRed*), (**E**) wt and (**E’**) mutant at 16 dpf. (**F**) Graphic presentation of the integrated density of fluorescence in the red channel in *tcf7l2* ^*exI*/*exI*^ mutant and wild-type sib controls in *Tg*(*ela3l:Crimson*) at 7 and 14 dpf. Data were obtained from 6 individuals per genotype, repeated in 2 different experiments. All reference to phenotypes was confirmed by genotyping. The integrated density was obtained using the Fiji software. Values represent the mean ± SEM. Asterisk above column indicate statistical differences among groups *p < 0.05.

To test the requirement of *tcf7l2* for indirect regulation of insulin expression in β cells, we compared the insulin expression in *tcf7l2* ^*exI*/*exI*^ homozygous mutants and *tcf7l2* ^+/+^ controls. When endogenous expression of *insulin* mRNA was analysed by whole-mount *in situ* hybridization at 9 dpf, we observed a significant diminution of *insulin* expression levels in *tcf7l2* ^*exI*/*exI*^ mutants, compared to wt siblings (Fig. 3A,A’). Hence, to gain further insights into the functional role played by tcf7l2, we examined *in vivo* the effects of its inactivation on the expression of endocrine and exocrine markers. The first step was the phenotypic characterization of *tcf7l2* mutants by using a transgenic zebrafish line that carries the *ins:DsRed* transgene, thus allowing to monitor *in vivo* both insulin reporter expression and β-cell number in *tcf7l2* mutants and their controls (Fig. 3B,B’). A quantitative analysis of insulin transgene expression, shown in Fig. 3C at 16 dpf, revealed a decreased signal in mutants compared to controls (Fig. 3C), confirming our observations on endogenous insulin expression. Although the amount of β cells in *tcf7l2* ^*exI*/*exI*^ mutants was not affected at early stages of development (shown at 7 dpf in Fig. 3D), a significant decrease of *ins:DsRed* positive cells was observed at 16 dpf in *tcf7l2* ^*exI*/*exI*^ homozygotes, compared with control sibs (16 dpf, Fig. 3D).

The alterations of the exocrine pancreatic morphology is frequently observed in T1D and T2D, and is referred as exocrine pancreatic insufficiency (EPI)^29^. However, the role of Tcf7l2 in the exocrine pancreas formation or maintenance is not known. Hence, we performed the phenotypic characterization of tcf7l2 mutants using a transgenic zebrafish line harbouring the *ptf1a:DsRed* transgene, an *in vivo* marker of pancreatic exocrine cells. As shown in Fig. 3E–E’, in *tcf7l2* ^*exI*/*exI*^ mutants both *ptf1a:DsRed* expression and exocrine pancreas morphology were altered, suggesting that in zebrafish, from the 16 dpf stage, *tcf7l2*-mediated activities significantly affect exocrine pancreas development. A quantitative analysis of *Tg*(*ela3l:E2Crimson*), an alternative exocrine marker, shown in Fig. 3F at 14 dpf, revealed a decreased signal in *tcf7l2* ^*exI*/*exI*^ mutants, confirming our observations on *Tg*(*ptf1a:DsRed*) at 16 dpf. The *ela3l* marker was not affected in mutants at 7 dpf (Fig. 3F).

### The adult pancreas of *tcf7l2*^*exI*/+^ heterozygous mutants is morphologically altered

As shown before, our analysis of pancreatic phenotypic defects at 16 dpf has been performed in *tcf7l2* ^*exI*/*exI*^ homozygous mutants. To study this phenotype in adults, we used *tcf7l2* ^*exI*/+^ heterozygous mutants, given that *tcf7l2* ^*exI*/*exI*^ homozygous fish are not viable from 6 wpf.

**Figure 4:**
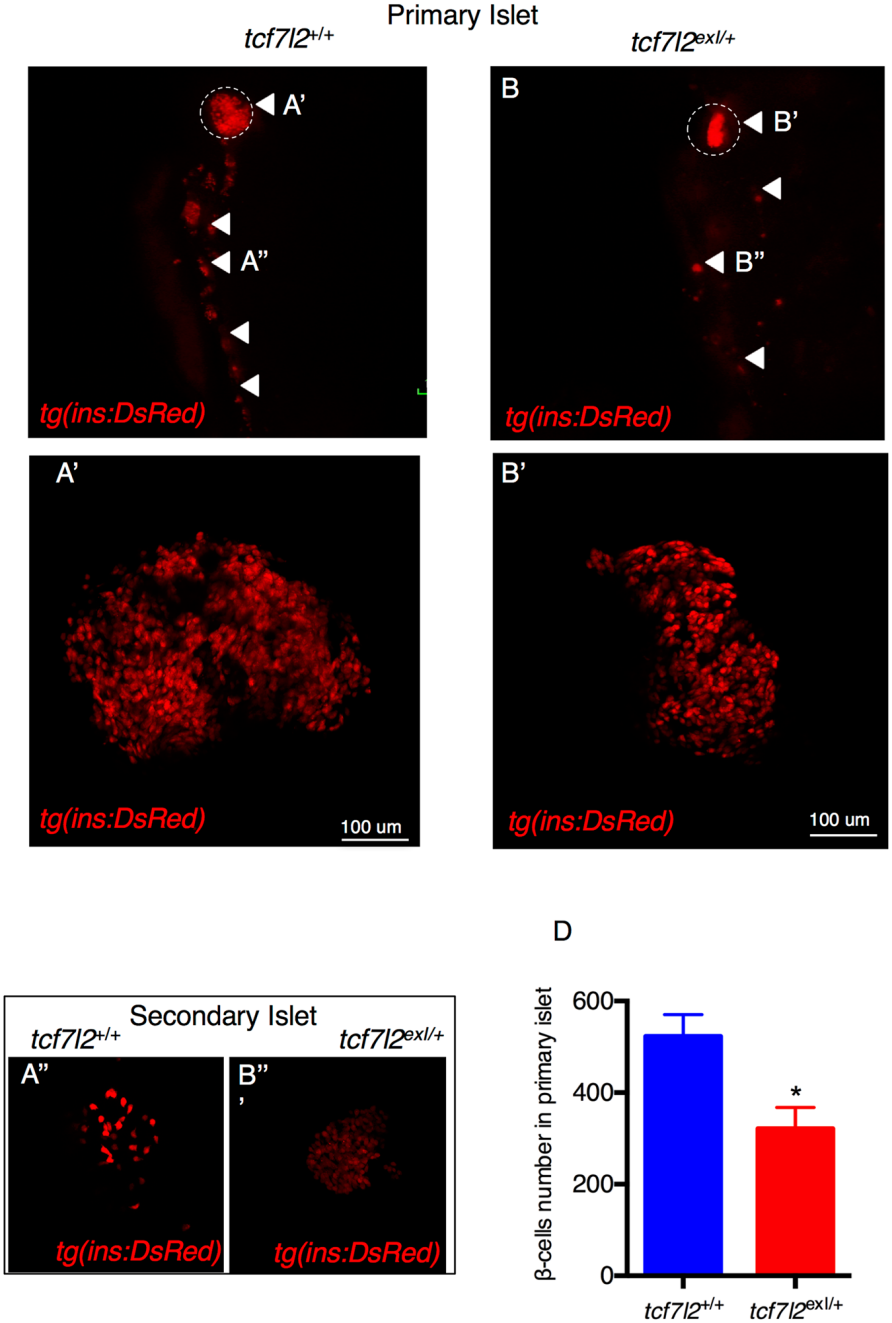
Morphology of β cells in wt and *tcf7l2* ^*exI*/+^ heterozygous adults. (**A**,**B**) Whole gut tissue extracted from 9 month-old wt and *tcf7l2* ^*exI*/+^ fish in *Tg*(*ins:dsRed*) background. Dashed circle: primary islet. Arrowheads: secondary islets and individual β cells extending caudally along the intestine. Examples of projection of a confocal stack image (**A’,B’**) of primary islets and secondary islet (**A”,B”**). (**C**) Quantification of β cells in 9 months old fish. Data were obtained from 3 individuals per genotype, repeated in 2 different experiments.

The crossing of *tcf7l2* ^*exI*/+^ heterozygotes with *Tg*(*ins:dsRED*) allowed us to study *in vivo* the effect of *tcf7l2* mutations on the adult pancreas morphology. Figure 4A and B show the endocrine pancreas in wild type and *tcf7l2* ^*exI*/+^ adults. Although the body size of *tcf7l2* ^*exI*/+^ heterozygous mutant was slightly smaller when compared with wt, morphometric analysis of the pancreas of *tcf7l2* ^*exI*/+^ zebrafish showed allometric reduction of overall organ size including the exocrine (not shown) and the endocrine compartment (Fig. 4A,B). Specifically, we could observe a reduction in the area of endocrine pancreas, with a decrease in the number of β cells (Fig. 4B’). A similar phenotype was present also in the secondary islets (4B”). To assess the phenotype of endocrine and exocrine pancreas in more detail, we performed histological analysis of pancreatic paraffin sections from 9-month-old adults, detecting more adipose tissue in mutant exocrine pancreas compared to wt (Supplementary Figure 2). This analysis confirmed that the pancreatic structure of *tcf7l2* ^*exI*/+^ mutants was more compact. No significant differences in the number of α cells were found between the *tcf7l2* ^*exI*/+^ and *tcf7l2* ^+/+^ (Supplementary Figure 3). These data indicate that *tcf7l2* might play a role in the control of β-cell structure and number, serving as an important regulator of gene expression and islet cell coordination.

**Figure 5:**
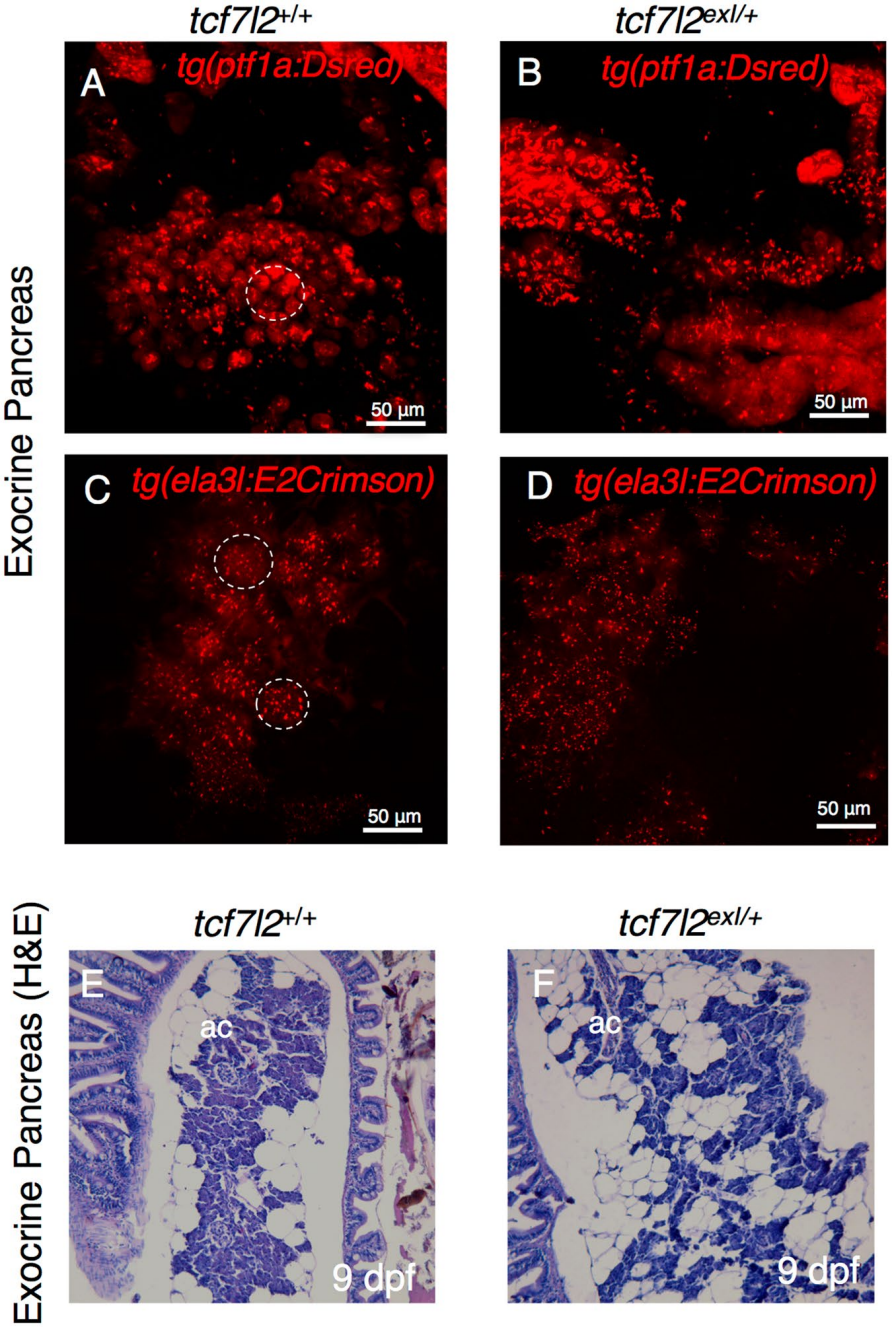
Morphology of exocrine pancreas in wt and *tcf7l2* ^*exI*/+^ heterozygous adults. (**A**,**B**) Projection of a confocal stack image of exocrine pancreas extracted from 9-month-old wt and *tcf7l2* ^*exI*/+^ fish in *Tg*(*ptf1a:DsRed*) background. (**C**,**D**) Projection of a confocal stack image of exocrine pancreas extracted from 6-month-old wt and *tcf7l2* ^*exI*/+^ fish in *Tg*(*ela3l:Crimson*) background. Dashed circles indicate typical acinar structures. Scale bar = 50 μm. (**E**,**F**) H&E staining of acinar cells (ac) of wt (**E**) and *tcf7l2* ^*exI*/+^ (**F**) at 9 mpf.

Given the developmental alterations displayed by the adult endocrine pancreas, we examined the role of *tcf7l2* in the exocrine compartment for possible defects. To address this question, we crossed the *Tg*(*ptf1a:DsRed*) and *Tg*(*ela3l:E2Crimson*) lines with *tcf7l2* ^*exI*/+^ mutants. As illustrated in Fig. 5A and B, in the *tcf7l2* ^*exI*/+^ adult mutants the acinar organization was difficult to discern. The elastase regulatory sequence drives highly specific expression of Crimson in the exocrine pancreas of both larvae and adults, allowing the observation of exocrine cell differentiation, proliferation, and morphogenesis *in vivo*. By confocal analysis of the wild types in *Tg*(*ela3l:E2Crimson*) background (Fig. 5C), the acinar cells and their lobular organization were clearly discerned, while in *tcf7l2* mutants (Fig. 5D) these histological features of exocrine tissue and acinar organization were not comparably organized, displaying acinar cells more dispersed and surrounded by abundant adipose tissue. We confirmed this arrangement of the exocrine tissue by histological analyses (Fig. 5E and F). The alteration of the morphology and organization of the exocrine tissue in mutants indicates a potential *tcf7l2*-specific function in exocrine tissue maturation.

### The role of vascular endothelium in pancreas development and β-cell regeneration

**Figure 6:**
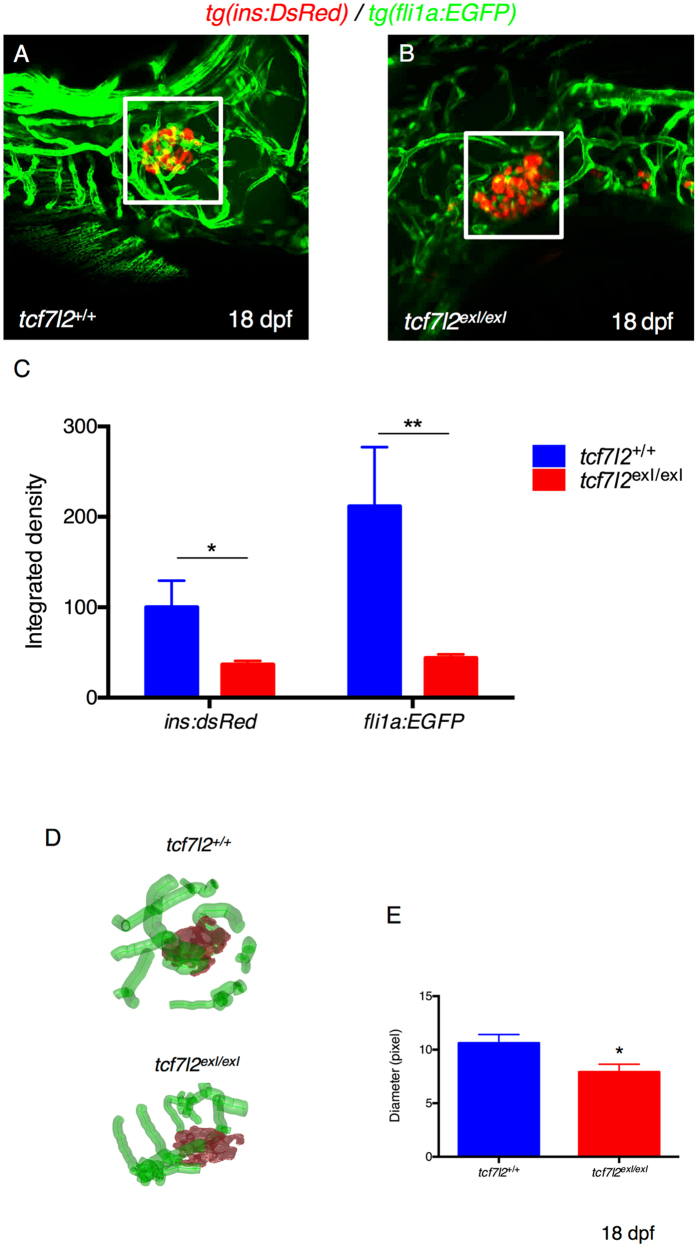
Defects in vascular endothelium and pancreas development of *tcf7l2* ^*exI*/*exI*^ homozygous larvae. (**A**,**B**) Analysis of pancreatic islet and blood vessels inside and around the pancreas of *tcf7l2* mutants and wild-type sib controls in *Tg*(*fli1a:EGFP*/*ins:DsRed*) background. Representative images were taken at 18 dpf by confocal microscopy at 20x magnification. (**C**) Graphic presentation of the integrated density of fluorescence in the red and green channel in *tcf7l2* ^*exI*/*exI*^ mutant and wild-type sib controls at 18 dpf. The regions for the analysis of integrated density of fluorescence are indicated by white boxes. Data were obtained using Fiji software. (**D**) Analysis and graphic presentation of vessels diameter; the mutant is characterized by decreased vessel diameter. Data were obtained from five individuals per genotype. All reference to phenotypes was confirmed by genotyping; *p < 0.05.

Wnt signalling and Tcf7l2 are fundamental in vascular development and, specifically, in endothelium formation^30^. Moreover, studies carried out in mouse and Xenopus have shown that, in the absence of vascular endothelial cells, Pdx1-positive endoderm cells fails to differentiate into β cells^31^. Endothelial cells are known to be extensively present in the pancreatic islet of zebrafish by approximately 52 hpf^24^, and we were able to clearly detect the first endothelial cells in the endocrine pancreatic primordium already at 30 hpf (Supplementary Figure 4A,B,﻿C). Thus, we reasoned that the role of Tcf7l2 in pancreas development could be mediated through the control of pancreatic vasculogenesis and the maintenance of pancreatic endothelium. To assess *in vivo* the possible role of Tcf7l2 in endothelial cells development and zebrafish pancreas formation, we analysed the vascular system in *tcf7l2* mutants by using a transgenic line expressing GFP under the control of the zebrafish *fli1a* promoter^28^. The Fli1 transcription factor is a highly-conserved member of the ETS-domain family of transcriptional activators and repressors. Transgenic *Tg*(*fli1:eGFP*)^*y1*^ zebrafish label the endothelial lineage, from angioblasts, during early development^32^ and during normal or defective pancreas development^24^. Hence, *tcf7l2* ^*exI*/+^ mutants were crossed with *Tg*(*fli1:eGFP*)^*y1*^. The analysis shows a decrease in the number and volume of pancreatic islet capillaries in *tcf7l2* ^*exI*/*exI*^ mutants (Fig. 6C). In particular, *tcf7l2* and wt zebrafish blood vessels of the islet region differ both in density and in the diameter of the capillaries, indicating that the pancreatic vascularization is clearly impaired in *tcf7l2* mutants (Fig. 6D).

To verify if this altered vascularization can underlie observed β-cell defects in *tcf7l2* mutants, we performed a drug-mediated perturbation of blood vessels and analysed its effects on the β-cell compartment. Specifically, we treated 2 dpf double transgenic embryos Tg(*kdrl:mCherry-ins:EGFP*) with the anti-angiogenic drug Pazopanib (as described in Li *et al*.^33^) (Supplementary Figure 4D,E). After three days of drug treatment, we clearly detected a reduction of *kdrl:mCherry*-positive small calibre vessels in the area surrounding the endocrine islet, in parallel with a significant decrease of *ins:EGFP* transgene expression, suggesting that a correct vascularization of the pancreatic islet is required for normal *insulin* expression in β cells.

Wnt signalling is extensively involved in tissue proliferation in multiple developmental contexts^17^; thus, in order to understand the possible contribution of *tcf7l2* to proliferation, mitosis was measured in mutant by DNA-incorporation assays using the thymidine analogue EdU. For the EdU incorporation assays, control animals and *tcf7l2* mutants were incubated with EdU at 6 dpf and analysed at 9 dpf. The overall image of the pancreatic region shows that the mutants have less EdU+ cells than controls, indicating that the proliferation rate is strongly reduced in mutants at 9 dpf (Supplementary Figure 5). Notably, a similar phenotype, with a loss of proliferation at the base of the intestinal folds of the middle and distal parts of the intestine, was observed by Muncan *et al*.^19^.

On the other way, to verify if, in parallel to the decreased proliferation, increased cell death could contribute to the pancreatic *tcf7l2* phenotype, acridine orange (AO) staining was performed at 11 dpf, detecting a larger amount of AO-positive dying cells in mutants compared to controls (Supplementary Figure 5).

As these are global analyses, performed on the whole organ, decreased proliferation or survival at the vascular level are not excluded. Thus, our findings indicate that the Wnt effector Tcf7l2 has a conserved function in formation, maintenance and proliferation of cells in the pancreatic region, suggesting that abnormalities in the generation of vessels may be a consequence of impaired proliferation/survival of endothelial cells, and that altered interaction between endothelial and endocrine cells could lead to anomalies in pancreas formation and function.

Since β-cell regeneration relies on nutritional factors delivered by the vasculature as well as on non-nutritional metabolic cues that rely on the systemic circulation, we sought to evaluate the β-cell mass during regeneration in *tcf7l2* mutants.

Wt and *tcf7l2* ^*exI*/*exI*^ embryos in *Tg*(*ins:NTR-mCherry*) background were treated with 7 mM Metronidazole (Mtz) starting from 4 dpf^23^. By 5 dpf (24 hr of treatment), the incubation of transgenic larvae with the pro-drug Mtz resulted in reduced β-cell mass. To investigate the regenerative capacity of the pancreatic islet after NTR/Mtz-mediated ablation, wt and *tcf7l2* ^*exI*/*exI*^ larvae were washed several times in fresh Mtz/DMSO-free medium and allowed to recover for 48 hr. Confocal imaging revealed that there is a quick and complete regeneration of β cells in the wt but not in *tcf7l2* ^*exI*/*exI*^ mutants (Supplementary Figure 6).

### Heterozygous *tcf7l2*^*exI*/+^ adults have a reduced rate of caudal fin regeneration at 7 mpf

**Figure 7:**
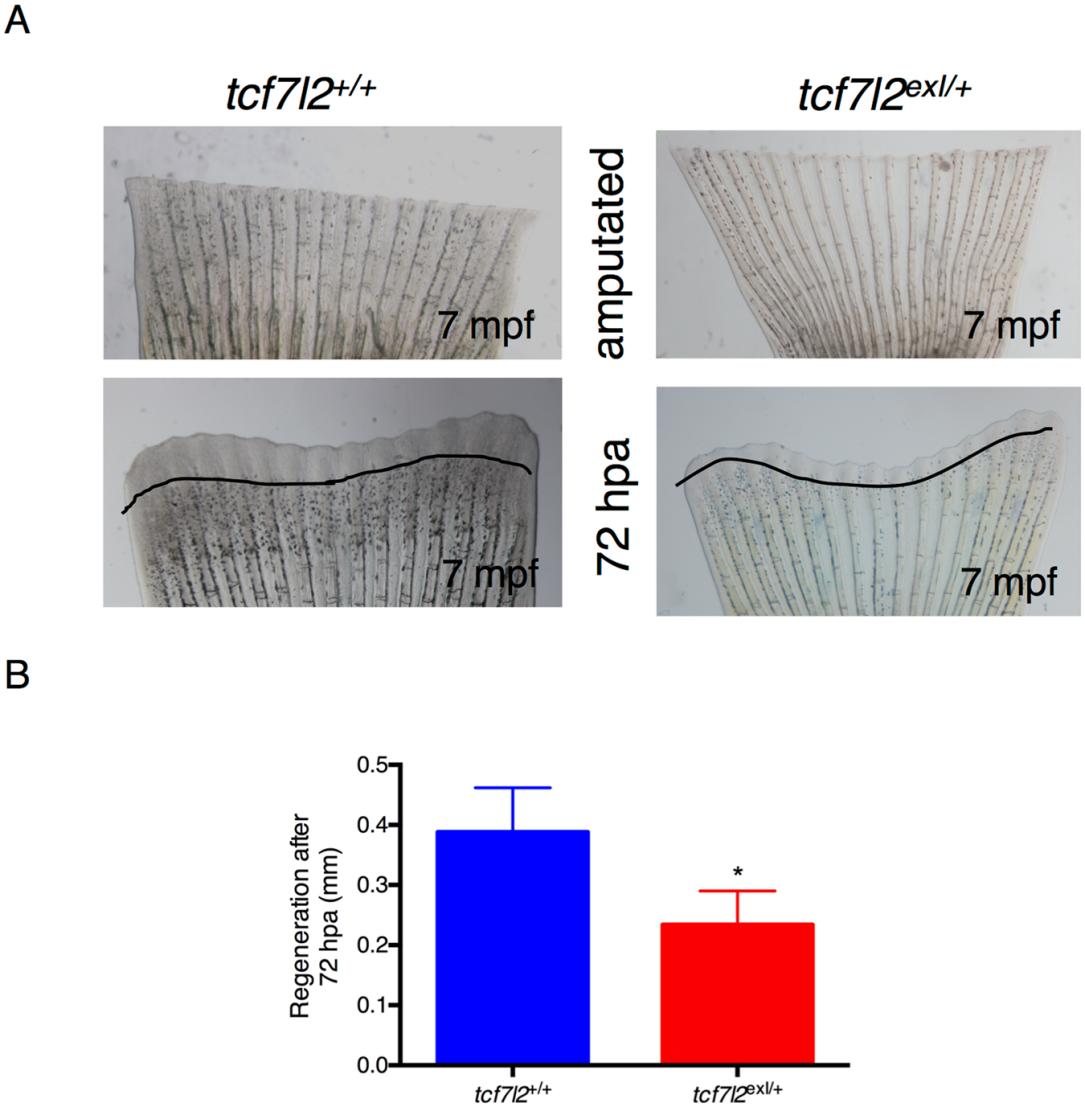
Heterozygous *tcf7l2* ^*exI*/+^ adults have a reduced rate of caudal fin regeneration. (**A**) Bright field live images of unamputated and regenerating fins in wild type and *tcf7l2* ^*exI*/+^ after 72 hours post-amputation. The area of regeneration was determined and the original cut line was used to normalize fin size differences. (**B**) Graphic presentation of the regeneration rate in controls and *tcf7l2* ^*exI*/+^ mutant fish. Data were obtained from six fishes per genotype, repeated in 3 different experiments.

In order to study the regenerative performances of *tcf7l2* mutants in other anatomical districts, the caudal fin of control and *tcf7l2* ^*exI*/+^ heterozygous individuals were amputated as previously described^34^. Images were collected at 72 hours post-amputation. The newly regenerated tissue was traced using Image J software, yielding the area of regeneration. In order to normalize for the differences in initial fin size, we divided this area by the dorso-ventral length of the fin at the amputation site. Tcf7l2 heterozygous zebrafish exhibited a statistically significant reduction compared to controls (Fig. 7B). Tissue regeneration requires the coordination of cell proliferation and apoptosis and we hypothesized that the reduction documented above could be due to alterations in either of these two processes. The results show that in *tcf7l2* ^*exI*/+^ mutants there is a decrease of the proliferative potential of the regenerating fin. A decrease in cell proliferation was also observed in *tcf7l2* ^*exI*/*exI*^ at 9 dpf by labelling S-phase nuclei with the click-iT EdU cell proliferation assay (Supplementary Figure 5C,D).

## Discussion

After the demonstration of a very strong association between the risk of T2D and specific polymorphisms in the *TCF7L2* gene^1^, this effector of Wnt signalling has started to draw global attention^4, 35^. Therefore, in this study our first goal has been to verify if mutations in the orthologous zebrafish gene could elicit a diabetic phenotype. To this purpose, we tested *tcf7l2* mutants for their blood glucose levels, detecting elevated post-prandial glucose levels and thus assessing that this mutant line can be a good model for Tcf7l2-dependent T2D.

In the literature, data show that individuals carrying T2D risk alleles at the *TCF7L2* locus, and those with T2D itself, have increased *TCF7L2* transcript levels^5^. However, in human islets both *TCF7L2* suppression^12^ and over-expression^5^ have been reported to cause impaired glucose-stimulated insulin secretion. These divergent effects raise concerns about the biological relevance of perturbing total *TCF7L2* transcript levels. T2D risk variants have been proposed to influence *TCF7L2* splicing patterns, raising the possibility that the ratio of functionally distinct mRNA isoforms, rather than the overall level of expression, could determine the pancreatic phenotype^36^.

These studies have directed our attention to the analysis of Tcf7l2 expression and Wnt signalling activity in zebrafish pancreatic islets, especially in the pancreatic β cells. Literature data present contrasting findings about Tcf7l2 expression in the mouse pancreas; notably, previous analyses have failed to detect Tcf7l2 by immunohistochemistry in mouse pancreatic islets^37^. In our study, the expression levels of *tcf7l2* and other members of the TCF/LEF gene family (*tcf7l1a*, *tcf7l1b*, *tcf7* and *lef1*), revealed by RNAseq in the zebrafish adult pancreatic cell types, indicate that *tcf7l2* is not expressed in β cells while it is present in δ cells and, at higher levels, in the acinar tissue. This finding has been further supported by observations with whole mount *in situ* hybridization that confirmed the expression of *tcf7l2* in the exocrine pancreas, intestine and liver. Overall, our analysis of *tcf7l2* expression in the zebrafish model confirms a pattern like the mouse system, but refines its distribution in the pancreatic islet, detecting signals in somatostatin-producing δ cells. As in mouse, our study also failed to detect signals in β cells, suggesting a non-cell autonomous effect of Tcf7l2 on β-cell function.

In T2D, β-cell insulin response to glucose is blunted. These changes are associated with structural and functional changes in pancreatic islets. Hence, β-cell dysfunction could be related to impaired insulin secretion due to many reasons, including insufficient β-cell mass, impaired regenerative capability and/or functional defects of the β cells, as well as structural/functional defects in surrounding tissues, such as the vascular and exocrine compartments.

*Tcf7l2* knockout mice or *Tcf7l2*/*Tcf7* double knockout mice have shown defects in gut development, but no abnormalities have been reported in pancreas development^38, 39^. The zebrafish *tcf7l2* null mutant shows clear morphological defects at 4 weeks post-fertilization and is not viable after 6 wpf^19^. The phenotype comprises a loss of proliferation at the base of the intestinal folds of the middle and distal parts of the intestine. In our study, we have detected decreased proliferation as well as increased cell death in the pancreatic region of zebrafish *tcf7l2* mutants, suggesting that both impaired tissue growth and reduced cell survival may underlie the observed pancreatic phenotypes. On the other way, the chronic elevation of blood glucose, that we have observed in the *tcf7l2* mutant, could have negative long-term effects on β-cell proliferation in the zebrafish as it does for mice^40^.

We have evaluated the regenerative capabilities of β cells in *tcf7l2* mutants, after tissue-specific induced damage. The zebrafish is a key genetic model system for vertebrate regeneration research; in this study we have taken advantage of available transgenic lines expressing nitroreductase (NTR) in β cells. NTR converts the pro-drug metronidazole to a toxin, resulting in β-specific cell death^23, 41^. With this ablation strategy, we could observe an impairment of β-cell regeneration in *tcf7l2* mutants. The limited regeneration of β cells found in heterozygous fish can be controlled by both systemic and local factors^42^; in other words, it can be a pancreatic-specific feature or a more general condition due to multi-tissue defects. The caudal fin is a suitable model for studying the molecular mechanisms regulating regeneration due to its accessibility and its relatively simple structure. In this study, we have examined caudal fin regeneration after amputation and confirmed that *tcf7l2* mutants are generally affected in their growth and regeneration capacities. Therefore, the *tcf7l2* mutant presented here appears as a valid tool to study diabetes and its regenerative complications.

We have also assessed if defects in the vascular compartment could be present in *tcf7l2* mutants, thus possibly contributing to the diabetic phenotype. Islets are indeed strongly vascularized, as their ability to sense blood glucose and release insulin depends on close contact with blood vessels; the failure of an adaptive response between blood vessels and β cells in the islet contributes to diseases such as T2D. Indeed, vascular pericytes are supporting cells of the islet vasculature that serve to regulate capillary blood flow and permeability, influencing changes in β-cell mass^43^. Interestingly, β cells secrete both pro- and anti-angiogenic factors that have been implicated in hyperglycaemia-induced maculopathy in adults^44^. In particular, angiogenic factors, including vascular endothelial growth factor (VEGF) are secreted from β cells, maintaining a dense and fenestrated capillary network; also, VEGF receptors are differentially expressed in islets versus acinar endothelium^31^.

We have observed vascular defects in *tcf7l2* mutant larvae with transgenic backgrounds *fli1:eGFP*/*ins:dsRED*, when analysed at 16 dpf (homozygous *tcf7l2* ^*exI*/*exI*^) and in adults (heterozygous *tcf7l2* ^*exI*/+^). Confocal microscopy of transgenic *ins:dsRED*/*tcf7l2* ^exI/exI^ mutants at 16 dpf shows a reduction in the number of β cells, and the analysis of transgenic *fli1:eGFP*/*ins:dsRED*/*tcf7l2* mutants indicates morphological abnormalities, with a statistically relevant reduction in the number and diameter of pancreatic islet capillaries.

If this effect of *tcf7l2* on blood vessels is indirectly exerted from adjacent tissues, or directly due to *tcf7l2* expression in vascular cells could not be fully clarified, due to vascular expression levels of *tcf7l2* undetectable by whole-mount *in situ* hybridization. However, based on our PCR amplification of *tcf7l2* transcripts in endothelial-enriched samples, a direct role of *tcf7l2* on the vascular compartment cannot be excluded. Interestingly, according to the Unigene database, human *TCF7L2* ESTs are also consistently detected in blood vessels. Moreover, our evidence of reduced insulin transgene expression, following vascular-specific perturbation, fully supports the hypothesis that mutations in *Tcf7l2* might affect β-cell function through the mediation of the vascular compartment.

We have also considered the exocrine tissue in our evaluation of the tcf7l2 phenotype. The analysis of adult tcf7l2^exI/+^ heterozygous individuals with transgenic backgrounds *Tg*(*ptf1a:DsRed*) or *Tg*(*ela3l:E2Crimson*) revealed a loss of acinar organization of the exocrine cells in the mutants. This could represent a consequence of the close anatomical, metabolic and functional links between the exocrine and endocrine pancreas, so that any disease affecting one of these parts will inevitably affect the other^29, 45^.

In conclusion, additional mechanistic studies are needed to fully evaluate different *Tcf7l2* models (zebrafish, rodents) and compare them with human patients in terms of compensation in the Lef/Tcf transcription factor family, genetic background and efficiency of gene ablation or inhibition. We should also consider the possibility that disease alleles may promote an imbalance of alternative splice forms of *TCF7L2*, thereby resulting in protein isoforms with opposing physiological effects^36^.

It is important to consider that *TCF7L2* is expressed in a broad spatial domain pattern, including tissues with important roles in glucose metabolism such as gut, brain, liver, skeletal muscle, fat, and bones^46^. This raises the possibility that, *in vivo*, TCF7L2 may not directly regulate glucose metabolism primarily through actions in β cells, but rather in tissues outside the endocrine islet, such as liver^11^, vasculature or exocrine pancreas.

As summarized in Table 1, we observed: (*a*) impaired glucose homeostasis in β cells deficient for *tcf7l2*; (*b*) an altered morphology of pancreas and pancreatic vessels; (*c*) defects in the regeneration of fins and β cells.

**Table 1:** Summary of detected phenotypes in heterozygous (*tcf7l2* ^*exI*/+^) and homozygous (*tcf7l2* ^*exI*/*exI*^) mutants.

	*tcf7l2* ^*exI*/+^	*tcf7l2* ^*exI*/*exl*^
Hyperglycaemia	✓7 mpf	
Defects in beta cells	✓9 mpf	✓16 dpf
Defects in exocrine pancreas	✓9 mpf	✓14 dpf
Defects in blood vessels		✓18 dpf
Altered regeneration of the fin	✓7 mpf	
Altered regeneration of beta cells	✓7 mpf	✓7 dpf

Tcf7l2 mutant phenotypes were detected at the indicated stages.

Prior studies provided evidence that pancreatic growth and differentiation are regulated by Wnt signalling^47–53^. However, these works did not present a mechanism for the *Tcf7l2*-mediated action. Our results collectively suggest that *Tcf7l2* is required in late steps of pancreas development, leading to a pleiotropic model that links *Tcf7l2* and T2D pathogenesis, with independent pancreatic (our study) and hepatic (other studies) contributions to the pathogenesis.

This zebrafish model of *TCF7L2*-linked dysfunction lends itself well to high throughput approaches, such as small molecule screens that can potentially be coupled with transgenic tools, including reporters of gene function, or β-cell ablation and regeneration. Such approaches open the chance for zebrafish to become an essential drug discovery and screening tool for the future of diabetes research.

## Material and Methods

### Zebrafish husbandry and transgenic lines

Animals were staged and fed as described by Kimmel *et al*.^54^. All animal experiments were performed in accordance with the European and Italian Legislations and with the specific permission for animal experimentation of the Local Ethics Committee. The project was examined and approved by the Ethics Committee of the University of Padua with protocol number 18746.

Tcf7l2 mutant carriers were identified as described in Muncan *et al*.^19^. For functional *in vivo* studies we used the following fish lines, that where crossed with the *tcf4hu892* or *tcf4* ^*exI*/*exI*^ mutant^19^: *Tg*(*fli1a:EGFP*)*y1* ^28^, *Tg*(*kdrl:mcherry*)*is5* ^55^, *Tg*(*ins:EGFP*)^56^, *Tg*(*7xTCF-Xla*.*Siam:GFP*)*ia4* ^26^, *Tg*(*ptf1a:EGFP*)*jh1*, *Tg*(*ptf1a:DsRed*)*ia6*, *Tg*(*gcga:GFP*)*ia1* ^25^ *Tg*(*ela3l:caspase*;*ela3l:E2Crimson*) (line abbreviated to *Tg*(*ela3l:E2Crimson*))^57^, *Tg*(*−1*.*2ins:DsRed*)^56^ referred to as *Tg*(*ins:DsRed*), and *Tg*(*ins:NTR-mCherry*)*ml10* (Dirk Meyer’s Lab) expressing a nitroreductase (NTR)-mCherry fusion protein in β cells. For β-cell ablation, the *Tg*(*ins:NTR-mCherry*)*ml10* line has been managed as described in Curado *et al*.^58^.

### Blood glucose measurement

Before postprandial analysis of glucose levels, each animal was fed with 25 mg of dry food (Tetramin Bioactive flakes, Tetra GmbH). Two different protocols for blood collection from zebrafish were used for the analysis of blood glucose.

In the first method, to obtain whole blood, fish were anesthetized with tricaine and ice-cold water and then cut cleanly through the pectoral girdle with scissors. The cut was immediately anterior to the articulation of the pectoral fin with the girdle, and severed the heart. Whole blood was analyzed immediately by applying a test strip directly to the cardiac blood. With this method, the quantity of blood collected depends on the size of the fish. We found that approximately 5 μl was typical, but as much as 10 μl was not uncommon as described in Eames *et al*.^20^.

The second method used is based on a non-lethal protocol for blood collection from zebrafish, as illustrated in Zang *et al*.^22^. Briefly, animals were tricaine-anesthetised, placed on a paper towel and gently dried. The needles were made from 1.0 mm-outer-diameter glass capillaries, pre-rinsed with 5 mg/ml heparin, and inserted in the tail region of the fish. The collected blood (0.5–1 ul) was placed on a piece of parafilm for measurement. After delicate pressure on the perforated skin area, the fish were transferred to a clean water tank and gently swirled for recovery.

The following glucose meters and test strips were used: Accu-Chek Aviva, for 0.6 μL samples (Roche Diagnostics), and GlucoMen LX sensor (Menarini Diagnostics), the latter suitable for blood volumes less than 0.5 μL.

### Expression levels of Tcf/Lef genes

Expression levels of *tcf7l2*, *tcf7l1a*, *tcf7l1b*, *tcf7* and *lef1* were obtained from RNAseq datasets prepared from purified mature pancreatic cells from adult zebrafish; these transcriptomic analyses will be presented in detail elsewhere (Tarifeño-Saldivia *et al*., manuscript in preparation). Briefly, the distinct endocrine cell types were prepared using the transgenic lines *Tg*(*insulin:GFP*) (created by Marianne Voz, unpublished line), *Tg*(*gcga:GFP*/*ins:mCherry*)*ia1* ^25^, and *Tg*(*sst2:GFP*)^59^ to isolate β, α and δ cells respectively. Acinar cells were isolated by using *Tg*(*ptf1a:GFP*)^60^. RNAs were purified from sorted cells and analysed by RNAseq. Sequenced samples were mapped to the zebrafish genome (Zv9, Ensembl genome version 75, http://www.ensembl.org) using the TopHat software^61^. ‘Per gene’ expression level was estimated using HTSeq-count^62^ and posterior normalization (library size) was performed using DESeq software^63, 64^.

### RNA extraction, reverse transcription and quantitative polymerase chain reaction (qPCR)

Total mRNA was isolated from different tissues of adult zebrafish using Trizol (Invitrogen, Carlsbad, CA), and 1 μg of total RNA reverse-transcribed using M-MLV Reverse Transcriptase RNase H- (Solis BioDyne).

qPCRs were performed in triplicate with EvaGreen method using a Rotor-gene Q (Qiagen) and the 5x HOT FIREPol ® EvaGreen® qPCR Mix Plus (Solis BioDyne) following the manufacturer’s protocol. The cycling parameters were: 95 °C for 14 min, followed by 45 cycles at 95 °C for 15 s, 59 °C for 35 s, and 72 °C for 25 s. Threshold cycles (Ct) and dissociation curves were generated automatically by Rotor-Gene Q series software. Sequences of specific primers used in this work are listed in supplementary material Table S1. Primers were designed using the software Primer 3 (http://bioinfo.ut.ee/primer3–0.4.0/input.htm). Sample Ct values were normalized with Ct values from zebrafish *elongation factor*-*1a* (*ef1a*) and *arp*.

### Whole-mount *in situ* hybridization

Embryos were staged according to Kimmel *et al*.^54^. Whole-mount *in situ* hybridizations on *tcf7l2* embryos and wt siblings were performed according to Thisse *et al*.^65^. The following digoxygenin- or fluorescein-labelled (Roche) antisense riboprobes were used: *insulin* ^66^ and *tcf7l2* R9 (kindly provided by H. Clevers). Control and mutant embryos have been hybridized in the same tube; control embryos had the tip of the tail cut. Following *in situ* hybridization, embryos were post-fixed in 4% buffered p-formaldehyde and mounted in 85% glycerol/phosphate-buffered saline (PBS) for microscope observation. Observations were made with a Leica DMR compound/Nomarski microscope and images were acquired with a Leica DC500 digital camera.

### Confocal analysis and vessel diameter measurements

Fluorescence was visualized under a Leica M165FC dissecting microscope and then with a Nikon C2 H600L confocal microscope. For *in vivo* analyses, embryos and larvae were anesthetised with tricaine and mounted in 1% low melting agarose gel. EGFP and mCherry fluorescence was visualized by using 488 and 561 nm lasers, respectively, through 20x and 40x immersion objectives (Nikon). All images were analyzed with Fiji software^67^.

Vessels were automatically segmented on the EGFP fluorescence image stack, by using a level set method (adapted from ref. 68) which takes into account the variability in the appearance of the vessel-related fluorescence. As long as the image intensity of the areas belonging to a vessel remains significantly higher than the background signal, it is considered as being part of a vessel. The methods incorporate three regularization parameters to make the estimation of abnormally large or irregular vessels unlikely.

The diameter at each point along a vessel centreline was estimated using a multi-stencil fast marching method (refs 69 and 70), which reliably computes the distance of a point from the object boundary.

With the same level set method^68^ used for segmenting the vessels, the β-cells boundaries were segmented on the mCherry fluorescence image stack.

A spherical region of interest was then drawn centred on the detected β-cell centroid and with a fixed radius of 0.1 mm.

All voxels belonging to a vessel centreline within this region have been considered, and their statistics measured: vessel density, mean diameter, and diameter standard deviation.

### EdU staining

To label proliferating cells in zebrafish, larvae were treated with EdU using the Click-iT Imaging Kit (Invitrogen C10085) adapted by S. Eckerle and J. Holzschuh^71^. At 6 dpf, larvae were incubated in EdU solution (10 mM EdU and 5% DMSO in fish water) for 25 minutes at RT to allow uptake of EdU into the embryos. The treated larvae were successively incubated at 28.5 °C for 3 days to allow the incorporation of EdU into the DNA of proliferating cells (S Phase). Samples were fixed in 4% PFA/1% DMSO for 2 h at room temperature (RT), washed in 1x PBST and de-yolked. EdU staining was performed as previously described^72^.

### AO staining

Cell death in whole zebrafish larvae was measured with vital dye acridine orange staining. Live embryos were immersed in 5 μg/ml acridine orange (Sigma), dissolved in PBS, in the dark for 30 min. Fluorescent signals were visualized with a Leica M165FC dissecting microscope, and images were acquired with a Leica DC7000 digital camera.

### Fin Regeneration

Fin regeneration studies were performed as previously described^73^. Zebrafish were anesthetized with tricaine and tail fins were cut with a scalpel immediately close to the proximal branch point of the dermal rays within the fin. Following amputation, fish were incubated at 28 °C for the indicated periods of time. The fish were again anesthetized, the regenerating fins were observed at 72 hours with a Leica M165FC dissecting microscope, and images were acquired with a Leica DC500 digital camera. The area of regeneration was divided by the dorso-ventral length of the fin to normalize the amount of regeneration for fish of different sizes.

### Larvae dissociation and fluorescence activated cell sorting (FACS)

Wild type *Tg*(*fli1a:EGFP*)^*y1*^ larvae at 6 dpf were dissociated as previously described (Zancan *et al*.)^74^ using 1x PBS, 0.25% trypsin phenol red free, 1 mM EDTA pH 8.0, 2.2 mg/ml Collagenase P (Sigma). Digestion was stopped by adding CaCl_2_ to a final concentration of 1 mM and fetal calf serum to 10%. Dissociated cells were rinsed once in PBS and resuspended in Opti-MEM (Gibco), 1% fetal calf serum and 1X Penicillin-Streptomycin solution (Sigma). Cells were filtered through a 40 μm nylon membrane and subjected to FACS (S3 Cell Sorter, Bio-Rad) with laser set at 488 nm and a 586/25 nm filter. GFP^+^ and GFP^−^ cells were separately collected in resuspension medium, and RNA was extracted using the RNA isolation kit Nucleospin® RNA XS (Macherey-Nagel).

### Statistical analysis

Statistical analysis was performed using Graph Pad Prism V6.0. Data are presented as the means ± SEM. The differences between the means were tested for significance using the non-parametric t-Student Test. A difference between two means was considered to be significant when p < 0.05 (*p < 0.05). Correction for multiple comparison (either using the over-stringent Bonferroni correction^75, 76^, or False Discovery Rate^77^ has not been performed. Given the sample size and exploratory nature of the study, we prefer to report possible false positive effects (Type II error, minimized through multiple comparison correction) than to exclude possibly relevant effects (Type I error, usually enhanced when applying multiple comparison correction).

## Electronic supplementary material

Supplementary Figures

Movie S4C
