# Supplementary material for: Tcf7l2 plays pleiotropic roles in the control of glucose homeostasis, pancreas morphology, vascularization and regeneration
Source: Sci Rep. 2017 Aug 29;7:9605. doi: 10.1038/s41598-017-09867-x (PMC5575064; doi:10.1038/s41598-017-09867-x)
Supplement: Supplementary file 1 — Supplementary Figures [file 41598_2017_9867_MOESM1_ESM.pdf]

Tcf7l2 plays pleiotropic roles in the control of glucose homeostasis, pancreas morphology, vascularization and regeneration

Nicola Facchinello, Estefania Tarifeño-Saldivia, Enrico Grisan, Marco Schiavone, Margherita Peron, Alessandro Mongera, Olivier Ek, Nicole Schmitner, Dirk Meyer, Bernard Peers, Natascia Tiso, Francesco Argenton

Supplementary Figure 1

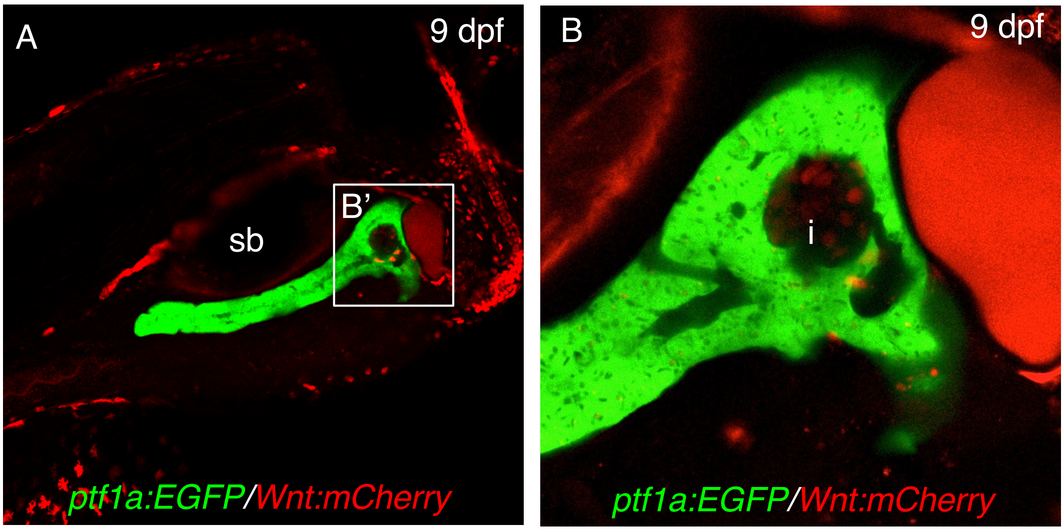

Supplementary 1. Visualization of Tcf/Lef-dependent expression using a responsive Wnt/b-catenin signaling-reporter transgenic zebrafish.
Analysis at 20x (A) and 40x (B) magnification of Wnt-reporter cells (red) in an exocrine pancreas-expressed transgenic line *(ptf1a:EGFP)* at 9 dpf. All figures are confocal Z-stack projections. The white square (B') in A indicates the region enlarged in B. sb: swim bladder, i: main endocrine pancreatic islet.

Supplementary Figure 2

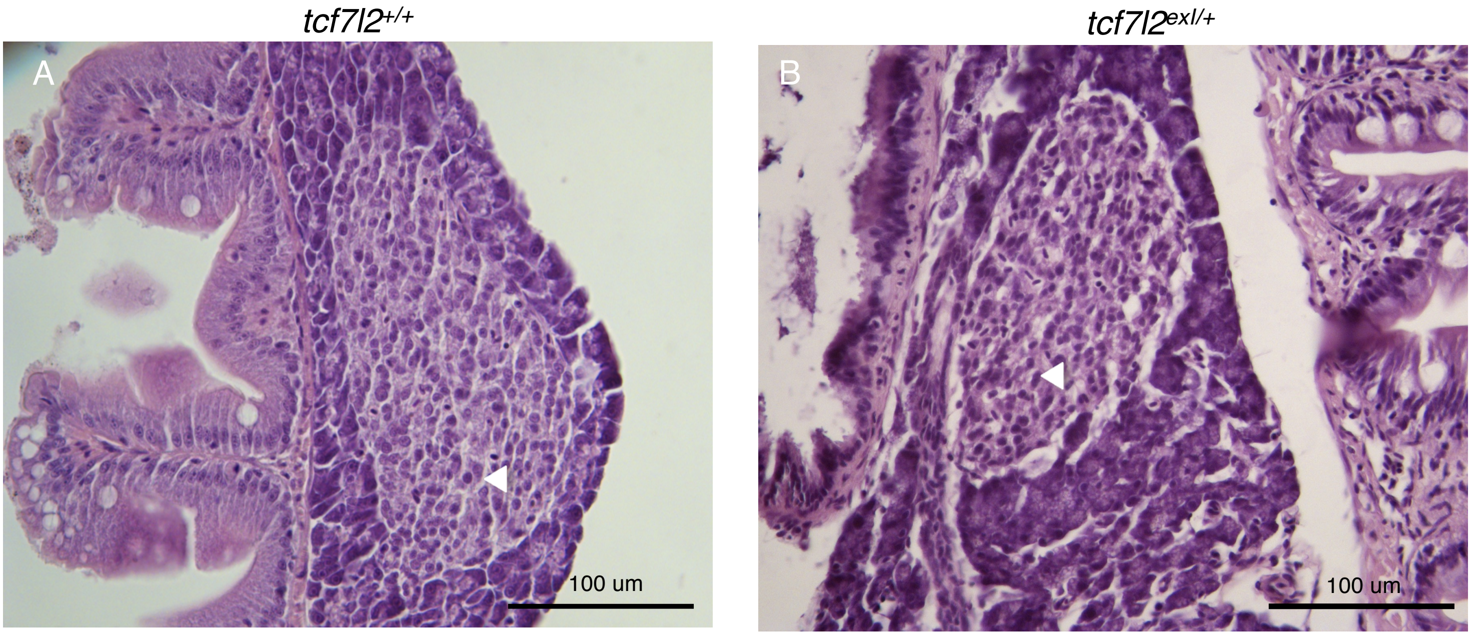

Supplementary 2. Histology of wild type and *tcf7l2* mutant adult pancreas

H&E staining of large principal islet of wt (A) and *tcf7l2*$^{exl/+}$ (B) at 9 mpf. White arrowheads point to beta cells.

Supplementary Figure 3

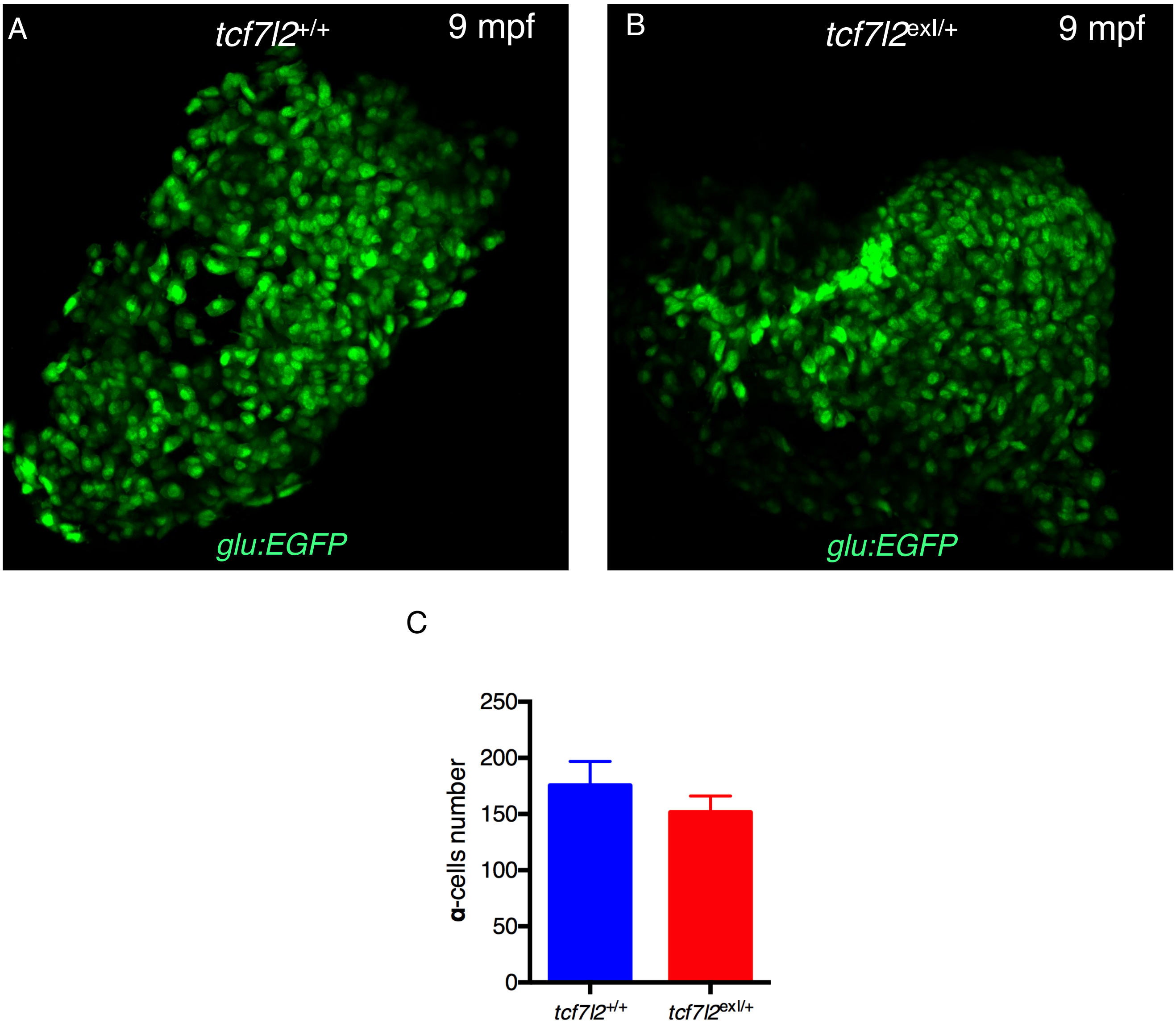

Supplementary 3. Impact of *tcf7l2* deletion on α-cell mass

Representative 2D images from wt and *tcf7l2*$^{exl/+}$ pancreata extracted from 9 month-old fish in α cell-specific *Tg(glu:eGFP)* background. (C) Quantification of α cells in 9-month-old fish. No significant difference between genotypes was observed.

Supplementary Figure 4

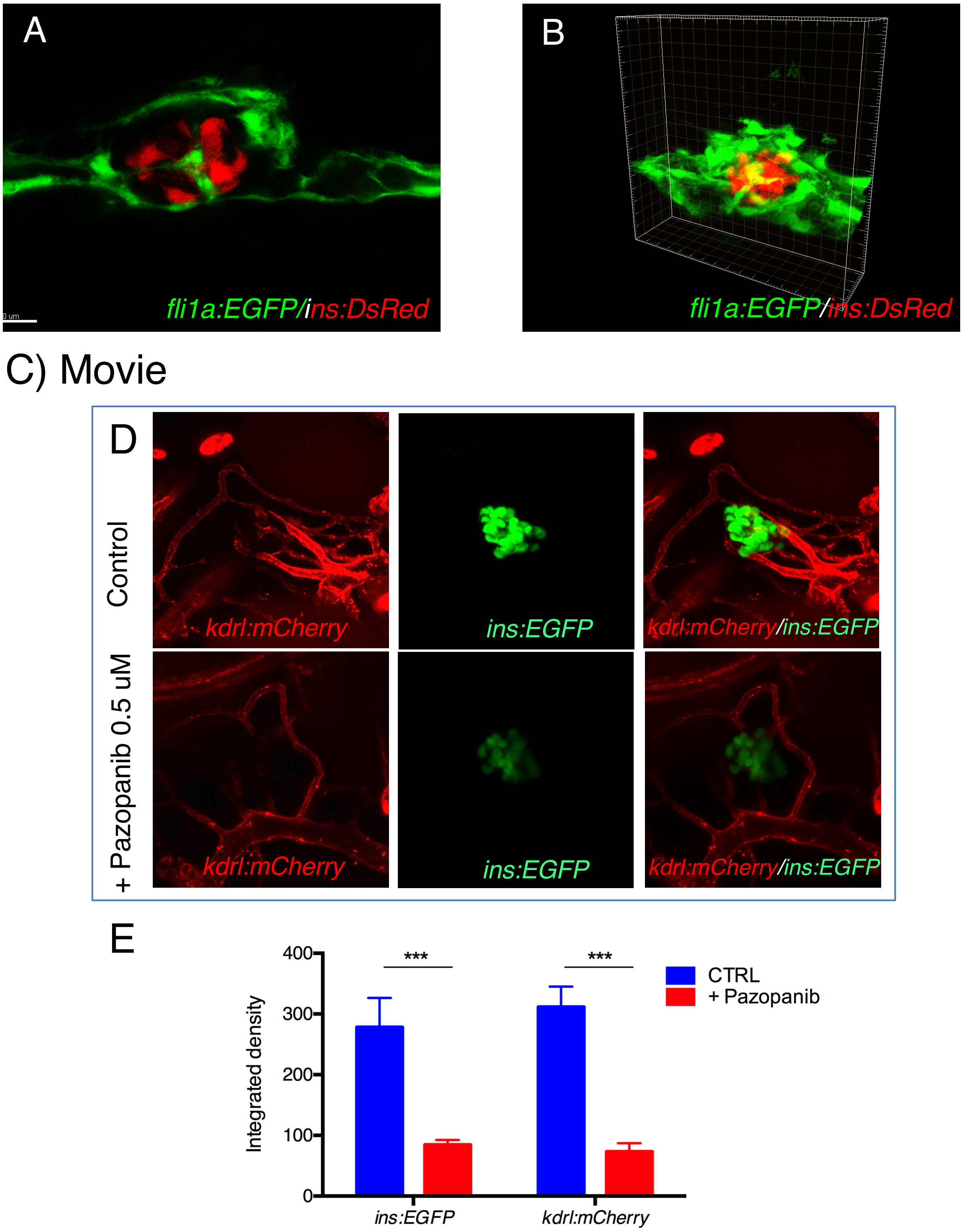

Supplementary 4. Imaging of pancreatic islet vascularization and treatment with Pazopanib

(A) Confocal section of a 30 hpf endocrine pancreas showing β cells (*ins:DsRed*) completely surrounded by blood vessels (*fli1a:EGFP*). Vessels are not only located in contact to the peripheral surface of the pancreatic islets, but also penetrate in the inner region between islets. (B) 3D reconstruction of a 30 hpf endocrine pancreas showing blood vessels wrapped around the β cells. C) Time-lapse video of a 30 hpf pancreatic endocrine islet (red, *ins:DsRed*) surrounded and penetrated by blood vessels (green, *fli1a:EGFP*). Full 3D stacks were taken every 10 minutes over a period of 3 hours. (D) Treated larvae were subjected to 0.5 μM Pazopanib treatment for 72 h (from 2 dpf to 5 dpf). (E) Integrated density analysis of fluorescence of 5 dpf zebrafish larvae with or without Pazopanib treatment. Values represent the mean ± SEM. Asterisks indicate that expression levels are significantly different from the control: *** $p<0.001$. n=6 larvae for each group.

Supplementary Figure 5

Click-iT EdU cell proliferation assay

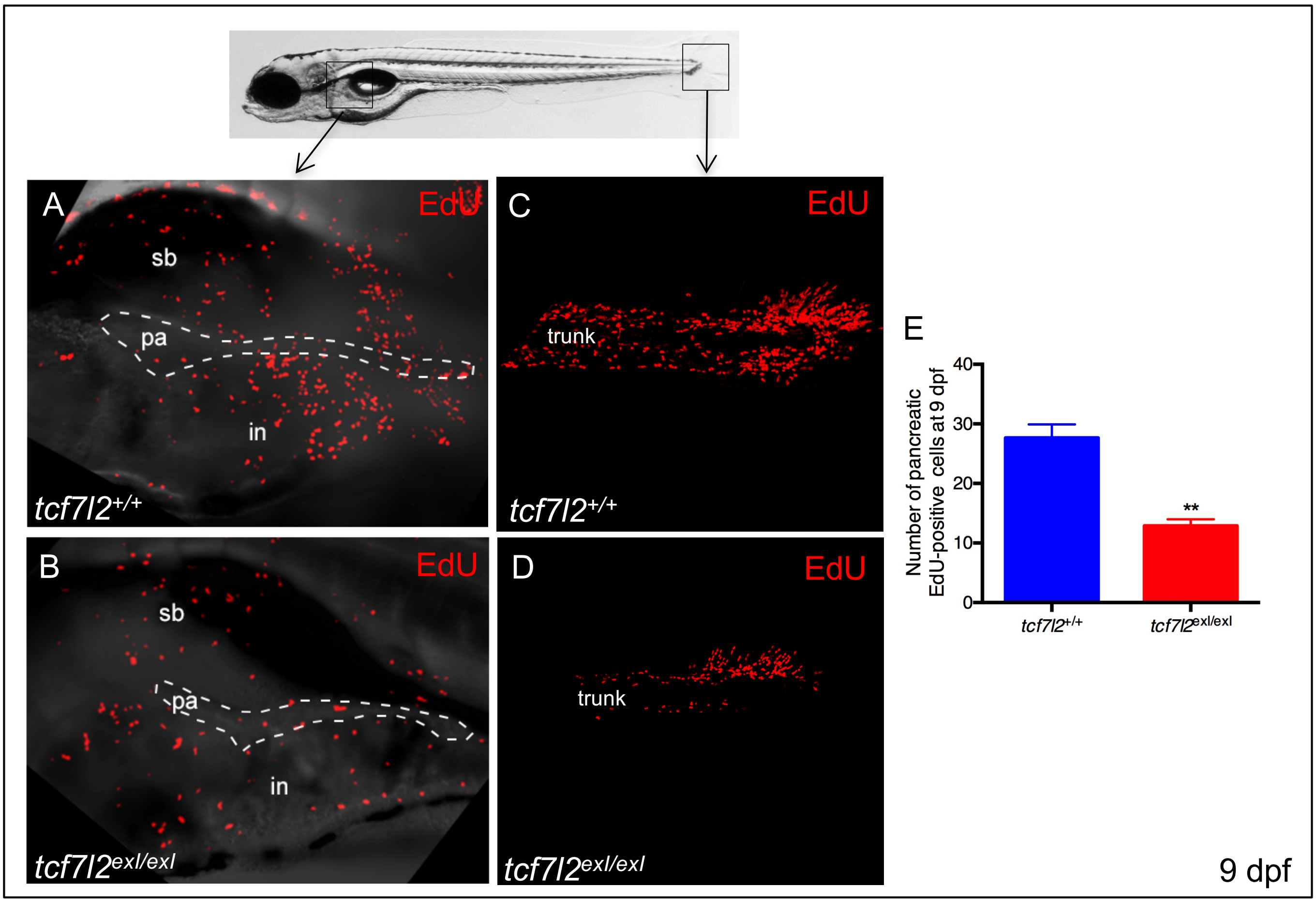

Acridine orange staining

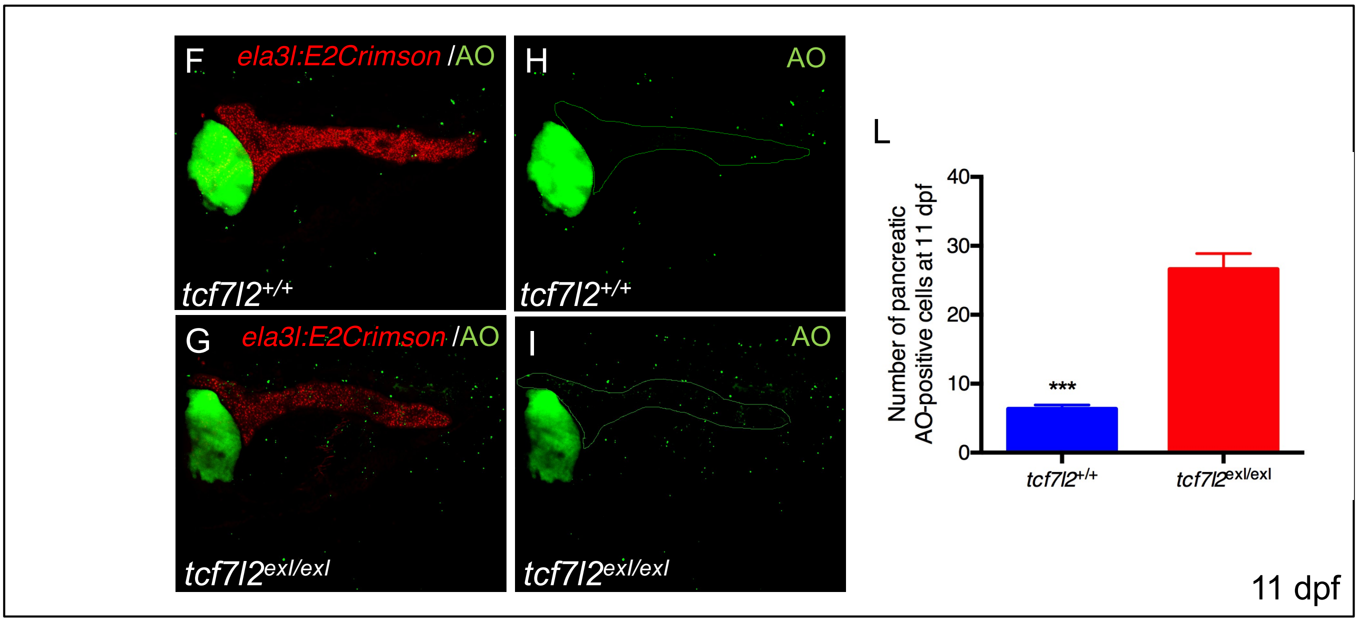

Supplementary 5. Decreased proliferation and increased cell death in *tcf7l2*$^{exl/exl}$

2D projections of confocal Z-series images of wild type (A) and *tcf7l2*$^{exl/exl}$ (B) at 9 dpf, showing EdU-positive cells (red) in the pancreatic region (dashed area). C, D: examples of caudal fins of wt and *tcf7l2*$^{exl/exl}$ mutant analysed by Click-iT EdU cell proliferation assay. Red signals indicate proliferating cells. F-I: Acridine Orange (AO, green dots). E,L: Quantification of EdU (E) and AO (L); n=6 for both charts. sb=swim bladder; pa=pancreas; in=intestine.

Supplementary Figure 6

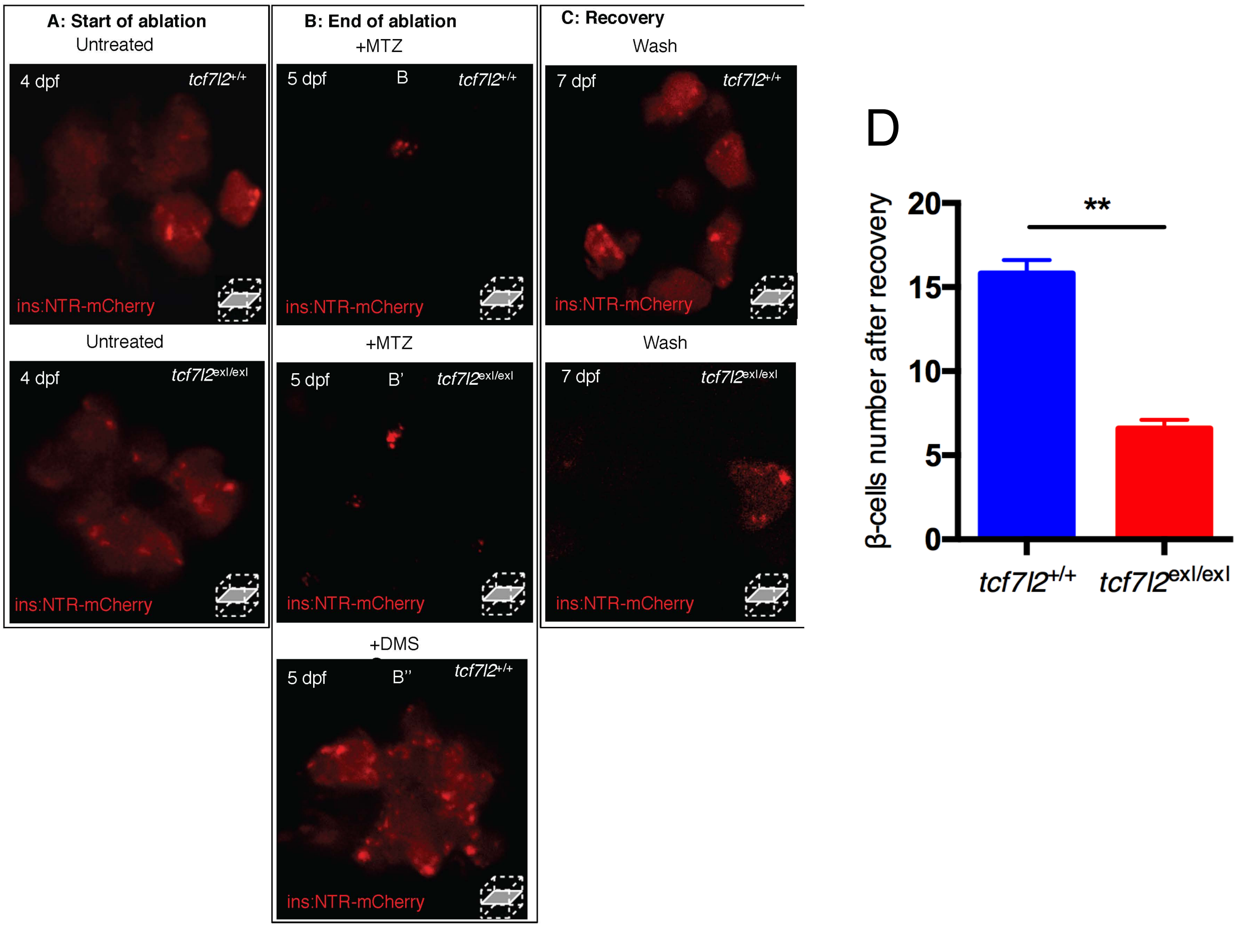

Supplementary 6. Impaired recovery of pancreatic β cells in *tcf7l2^exl/exl^* mutants

Confocal microscopy was used to monitor the progression of ablation in *tcf7l2*$^{exl/exl}$ and wt in *Tg(ins:NTR-mCherry)* larvae throughout their treatment with DMSO or Mtz.

Control and mutant larvae at 4 dpf before treatment (A), at 5 dpf, after treatment for 24 h (B) with 7 mM Mtz (B,B') or DMSO (B''), and at 7 dpf, after 48 h recovery (C). Loss of mCherry in treated individuals indicates that β cells have been successfully ablated; (C) fluorescence levels indicate cell recovery in the wt but not in the *tcf7l2*$^{exl/exl}$ mutant.

(D) Quantification of the number of β cells after recovery of *tcf7l2*$^{exl/exl}$ and control siblings. Data were obtained from six individuals per genotype. All reference to phenotypes was confirmed by genotyping. Values represent the mean ± SEM. Asterisk above column indicate statistical differences among groups ** $p<0.01$.

Primer	Sequence
ef1a-F	5'-GACAAGAGAACCATCGAG-3'
ef1a-R	5'-CCTCAAACTCACCGACAC-3'
tcf7l2-F	5'-CCTCCGCCTAGATCTGAAAG-3'
tcf7l2-R	5'-GGTCGGAGAAAGCGATCC-3'
egfp-F	5'-CCGACCACATGAAGCAGCAC-3'
egfp-R	5'-CCAGGATGTTGCCGTCCTC-3'
arp-F	5'-CTGAACATCTCGCCCTTCTC-3'
arp-R	5'-TAGCCGATCTGCAGACACAC-3'

Supplementary Table 1. List of primers used for qPCR.
